# Restricted dog leucocyte antigen (DLA) class II haplotypes and genotypes in Beagles

**DOI:** 10.1016/j.tvjl.2014.12.032

**Published:** 2015-03

**Authors:** Francesca Soutter, Lorna J. Kennedy, William E.R. Ollier, Laia Solano-Gallego, Brian Catchpole

**Affiliations:** a Department of Pathology and Pathogen Biology, Royal Veterinary College, North Mymms, Hertfordshire AL9 7TA, United Kingdom; b Centre for Integrated Genomic Medical Research, University of Manchester, Stopford Building, Oxford Road, Manchester M13 9PT, United Kingdom; c Departament de Medicina i Cirurgia Animal, Facultat de Veterinaria, Universitat Autònoma de Barcelona, Barcelona, Spain

**Keywords:** Beagle, Dog leucocyte antigen, Major histocompatibility complex

## Abstract

Beagles are commonly used in vaccine trials as part of the regulatory approval process. Genetic restriction within this breed and the impact this might have on vaccine responses are rarely considered. This study was designed to characterise diversity of dog leucocyte antigen (DLA) class II genes in a breeding colony of laboratory Beagles, whose offspring are used in vaccine studies. DLA haplotypes were determined by PCR and sequence-based typing from genomic DNA extracted from blood. Breeding colony Beagles had significantly different DLA haplotype frequencies in comparison with pet Beagles and both groups showed limited DLA diversity. Restricted DLA class II genetic variability within Beagles might result in selective antigen presentation and vaccine responses that are not necessarily representative of those seen in other dog breeds.

## Highlights

• Dog leucocyte antigen (DLA) class II genes were examined in laboratory Beagles.
• Laboratory Beagles represent a different immunogenetic pool compared with pet Beagles.
• Beagles do not express the full range of DLA haplotypes seen in the canine population.
• Beagles could be suitable for vaccine studies if genetic diversity is maintained.

Vaccine efficacy is fundamental to ensuring protection of animals against infectious disease and in maintaining herd immunity. Beagles are used for vaccine trials, designed to provide evidence of efficacy for regulatory approval. However, dog breeds, including the Beagle, have been derived from at least two genetic bottlenecks (Lindblad-Toh et al., 2005), which might affect their immunogenetic diversity and vaccine responses. Vaccination induced antibody titres can vary with breed, although genetic factors have yet to be identified (Kennedy et al., 2007b).

Dog leucocyte antigen (DLA) class II genes encode major histocompatibility complex (MHC) molecules that are involved in antigen presentation to CD4^+^ T cells. In dogs, these consist of three, highly polymorphic, loci, DLA-DRB1, DLA-DQA1 and DLA-DQB1 (Wagner, 2003). There is a large degree of inter-breed variability, but often limited intra-breed diversity, of DLA haplotypes (Kennedy et al., 2002a). The aim of the present study was to characterise DLA haplotypes in laboratory Beagle dogs and to compare their DLA diversity with a pet Beagle population.

Residual blood samples were obtained from laboratory Beagles (*n* = 100) in a commercial breeding facility, that provided puppies for vaccine trials in Europe; samples were collected for another study (project number 114-C-E-01-10; date of Animal Care and Ethics Committee approval 7 June 2010). Blood samples from pet Beagles (*n* = 47) were obtained from the Royal Veterinary College genetic archive, containing residual samples following completion of diagnostic testing with informed owner consent and with approval in 2004 from the institutional Ethics and Welfare Committee. Forty-eight pet Beagles, previously genotyped at the University of Manchester (date of Animal Care and Ethics Committee approval April 2004) were also analysed and included in the pet Beagle group. Information on pedigrees and relatedness of the dogs was not available.

Genomic DNA was extracted using the GenElute Blood Genomic DNA Kit (Sigma-Aldrich). PCR was performed in 25 µL reactions, with 1 µL DNA as template, DLA-specific primers (2 µL at 20 pmol/µL final concentration; Sigma-Aldrich) (see Appendix: Supplementary Table S1), 5 µL Hi-Spec additive, 2.5 µL ImmoBuffer, 1.25 µL MgCl_2_ (2.5 mM final concentration), 0.25 µL deoxynucleotide triphosphates (1 mM final total concentration) and 0.1 µL (1.25 IU) Immolase DNA polymerase (Bioline). PCR was performed using a G-Storm GS1 Thermal Cycler (Gene Technologies) at 95 °C for 10 min, followed by 35 cycles of 94 °C for 40 s, 55 °C for 30 s for DQA1 or 60 °C for DRB1 and DQB1, and 72 °C for 1 min, then 72 °C for 10 min. PCR products were processed using the GenElute PCR Clean-up Kit (Sigma-Aldrich) and submitted for sequencing (Source Bioscience) using primer M13F. DLA analysis and allele assignment was performed using SBT Engine version 2.17 (GenDx). Haplotype frequencies were compared between groups using Fisher's exact test (Monte Carlo method) in SPSS (PASW Statistics 18, IBM).

Fourteen DLA haplotypes were identified (12 in pet Beagles and eight in laboratory Beagles). Only four haplotypes occurred at a frequency >0.05 in each group and three of these more frequent haplotypes were the same in both groups (Table 1). Whilst some overlap existed in haplotypes between the two groups, the laboratory Beagle group demonstrated a significant difference in DLA haplotype frequencies compared with pet Beagles (*P* < 0.01). More pet Beagles were homozygous (frequency = 0.45) than laboratory Beagles (frequency = 0.21; χ^2^ = 11.93; *P* < 0.001). Pet Beagles were predominantly homozygous for haplotype DLA-DRB1*001:02–DQA1*001:01–DQB1*002:01 (frequency = 0.70 of homozygous pet Beagles; *n* = 43).

These results show that the laboratory Beagles had a different DLA profile, compared with the pet Beagles. Founder effects, selective breeding within a closed gene pool and use of popular sires have had an impact on genetic diversity within pedigree breeds (Lindblad-Toh et al., 2005; Calboli et al., 2008). The difference between these populations could be the result of selective breeding practices, since the groups have been bred for different purposes and likely represent subpopulations from historical Beagle stock with distinct founders (Kennedy et al., 2002b).

Neither group represents the DLA diversity in the dog population as a whole, where some breeds (e.g. Jack Russell terrier) express a more varied DLA profile, whereas others (e.g. Rottweiler) are even more restricted (Kennedy et al., 2007a). The limited diversity of DLA types could influence vaccine responses in Beagles and a product tested in Beagles will not necessarily perform similarly in other dog breeds.

Both groups were relatively restricted in DLA haplotype diversity. Whilst this restriction is not as marked as for some breeds, such as the Rottweiler, where only four haplotypes have been identified (Kennedy et al., 2002a), it is likely to affect the repertoire of peptide epitopes presented to CD4^+^ T cells upon antigenic stimulation. Since some haplotypes are only represented in a small number of Beagles, there is a risk that their frequency may diminish in subsequent generations if breeding strategy is not considered within a closed colony.

There was a significant difference in DLA homozygosity between groups. Without pedigrees or breeding strategy information, we can only hypothesise that this high frequency of homozygosity within the pet Beagles might indicate a higher degree of inbreeding and/or a potential dominant sire effect. Homozygosity appears to vary between dog breeds, depending on the size of the population base; a previous study found 28.4% homozygosity across breeds (Kennedy et al., 2002a).

Laboratory Beagles represent a different immunogenetic pool compared with pet Beagles. They may be a suitable choice for vaccine studies if genetic diversity and heterozygosity can be maintained by strategic breeding. However, laboratory Beagles do not express the full range of DLA haplotypes and thus are not representative of the diversity seen within the pet dog population as a whole. This poses potential problems for the use of any one breed in vaccine studies, since DLA type could influence responses to antigenic challenge. MHC class II heterozygosity in humans has been associated with enhanced resistance and more effective clearance of pathogens (Thursz et al., 1997; Carrington et al., 1999). Further studies are needed to investigate the impact of DLA genotype on susceptibility to infectious disease and response to vaccination in dogs.

## Conflict of interest statement

None of the authors of this paper has a financial or personal relationship with other people or organisations that could inappropriately influence or bias the content of the paper.

## Figures and Tables

**Table 1:** Frequencies of three DLA locus haplotypes in pet and laboratory Beagles.

Haplotype			Pet Beagles (*n* = 95)			Laboratory Beagles (*n* = 100)			Fisher's exact test *P* value
DRB1	DQA1	DQB1	Number of homozygous dogs	Total number of haplotypes	Haplotype frequency (%)	Number of homozygous dogs	Total number of haplotypes	Haplotype frequency (%)	
001:02	001:01	002:01	30	87	45.8	2	34	17	<0.01
015:01	009:01	001:01	6	36	18.9	5	50	25	NS
001:01	001:01	002:01	2	21	11	8	56	28	<0.01
002:01	009:01	001:01	1	17	8.9	0	7	3.5	<0.05
008:01	003:01	004:01	1	9	4.7	0	0	0	<0.01
006:01	005:01	007:01	1	8	4.2	6	46	23	<0.01
001:02	009:01	001:01	1	5	2.6	0	0	0	<0.05
006:01	004:01	013:03	1	2	1	0	0	0	NS
015:01	006:01	003:01	0	2	1	0	0	0	NS
002:01	009:01	002:01	0	1	0.5	0	2	1	NS
008:02	001:01	002:01	0	1	0.5	0	0	0	NS
020:01	004:01	013:03	0	1	0.5	0	0	0	NS
015:01	006:01	020:02	0	0	0	0	4	2	NS
048:01	004:02	023:01	0	0	0	0	1	0.5	NS
Total			43	190	100	21	200	100	

NS, not significant (*P* ≥ 0.05).

## Appendix Supplementary material

Table S1 Oligonucleotide primers used in the study.
