# Supplementary material for: Restricted dog leucocyte antigen (DLA) class II haplotypes and genotypes in Beagles
Source: Vet J. 2015 Mar;203(3):345–7. doi: 10.1016/j.tvjl.2014.12.032 (PMC4366010; doi:10.1016/j.tvjl.2014.12.032)
Supplement: Table S1 — Oligonucleotide primers used in the study. [file mmc1.docx]

**Appendix: Supplementary Table 1**

Oligonucleotide primers used in the study.

| Primer | Direction | Primer sequence | Amplicon size  (base pairs) |
| --- | --- | --- | --- |
| DLA-DRB1 | Forward | 5’-CCGTCCCCACCAGCACATTTC-3’ | 270 |
|  | Reverse | 5’-TGTAAAACGACGGCCAGTGTCACACACCTCAGCACCA-3’ |  |
| DLA-DQA1 | Forward | 5’-TGTAAAACGACGGCCAGTCTCAGCTGACCATGTTGC-3’ | 243 |
|  | Reverse | 5’-GGACAGATTCAGTGAAGAGAG-3’ |  |
| DLA-DQB1 | Forward | 5’-TGTAAAACGACGGCCAGTCTCACTGGCCCGGCCTGTCTC-3’ | 267 |
|  | Reverse | 5’-CACCTCGCCGCTGAACGTG-3’ |  |
| M13F | Forward | 5’-TGTAAAACGACGGCCAGT-3’ |  |

The M13F primer-binding region used in the sequencing reaction is underlined.
